# Percutaneous Coronary Intervention in Heart Failure: Knowledge Gaps and Opportunities

**DOI:** 10.1016/j.jscai.2022.100032

**Published:** 2022-04-11

**Authors:** Behnam N. Tehrani, Abdulla A. Damluji, Wayne B. Batchelor

**Affiliations:** a Inova Heart and Vascular Institute, Inova Health System, Falls Church, Virginia; b Division of Cardiology, Johns Hopkins University School of Medicine, Baltimore, Maryland; c Division of Cardiology, Duke University School of Medicine, Durham, North Carolina

**Keywords:** Percutaneous coronary intervention, heart failure, coronary artery disease, myocardial recovery, frailty

“Opportunity is often Delivered in a Fog of Uncertainty”

Jon Kabat-Zinn

Six million adult Americans live with heart failure (HF), and the prevalence is expected to increase by 46% by the year 2030.^1^ Two-thirds of all HF diagnoses are primarily ischemic in origin.^2^ While coronary artery bypass graft (CABG) surgery has been demonstrated to improve survival in patients with HF and coronary artery disease (CAD), less than 10% ultimately undergo surgical revascularization.^3^ Potential explanations for this finding include the following: 1) increasingly older patients presenting with complex age-related comorbidities; 2) clinical and coronary anatomic complexities such as diffuse disease, heavy coronary calcification, long lesions, and/or poor native vessel targets for bypass grafting; and 3) physiologic complexities related to systolic and diastolic HF and hemodynamic derangements, including pulmonary hypertension and cardiogenic shock.^4^ As a result, an increasing number of patients with HF and CAD are referred to multidisciplinary heart teams for “complex high-risk (and indicated) percutaneous coronary intervention” (PCI).^4^ While advances in hemodynamic support and interventional techniques have afforded us a higher likelihood of technical success and more complete revascularization, the role of PCI in patients with HF due to impaired left ventricular systolic function remains unclear, particularly in the absence of randomized clinical trials proving its superiority or noninferiority compared to CABG and/or medical therapy.^2^^,^^5^

In this issue of the *Journal of the Society for Cardiovascular Angiography and Interventions,* Ahmad et al.^6^ provide a comprehensive review of the current evidence for PCI in HF. Observational studies comparing PCI to CABG in HF, strategies to achieve optimal PCI results, the impact of complete revascularization on clinical outcomes, the role of viability testing, and the emergence of advanced mechanical circulatory support devices to facilitate complex high-risk (and indicated) PCI are reviewed. They highlight several unanswered questions in the field and burgeoning research studies which will better inform patient care. The review appropriately highlights the remarkable absence of any randomized controlled clinical trial evaluating PCI in this setting. However, additional gaps in knowledge remain that merit further discussion and investigation (Table 1). For example, what is the influence of nonobstructive CAD on outcomes in patients with HF? Classically, ischemic cardiomyopathy has been defined as HF in patients with prior myocardial infarction (MI), PCI or CABG, or ≥75% luminal stenosis in ≥2 epicardial vessels.^7^ Little is known, however, about the prognosis of patients with HF with nonobstructive CAD compared to those with no angiographic CAD, 2 cohorts that have historically been categorized as “nonischemic.” Is the mere presence of coronary atherosclerosis in the setting of HF an “innocent bystander” or is it a harbinger of future adverse events? A contemporary analysis of 12,814 patients with left ventricular ejection fraction (LVEF) <35% from the Canadian CorHealth Cardiac Registry (2010 to 2015) reported an 82% increased risk of CV death and an 18% incremental risk of all-cause death in patients with nonobstructive CAD compared to patients without angiographic CAD.^8^ However, interestingly there was no difference in the risk of acute MI, HF, or stroke,^8^ which runs counter to contemporary beliefs that implicate nonobstructive vulnerable plaque as the putative cause of acute ischemic events. There is also increasing recognition of the role coronary microvascular dysfunction plays in mediating myocardial ischemia in patients with HF due to both reduced and preserved ejection fraction who also have nonobstructive CAD.^9^^,^^10^ These observations highlight the need for better risk stratification and more effective targeted therapies for the varying phenotypes of CAD and microcirculatory dysfunction that coexist with HF.^2^

**Table 1:** Existing and potential research opportunities for PCI in ischemic cardiomyopathy

Clinical gaps in knowledge	Study designs
1. PCI in acute HFrEF without CS	RCT
	1. STEMI-DTU (pVAD + PCI vs. PCI)
	(NCT03947619)
2. PCI in acute HFrEF with CS	RCT
	1. DanGer Shock (pVAD + PCI vs PCI)
	(NCT01633502)
	2. ECLS-SHOCK (ECMO + PCI vs PCI)
	(NCT03637205)
	3. EURO-SHOCK (PCI + Early ECMO vs. PCI)
	(NCT03813134)
	4. ANCHOR (ECMO + IABP vs SOC)
	(NCT04184635)
3. PCI in chronic HFrEF without CS	RCT
	1. PROTECT IV (pVAD + HR-PCI vs. SOC PCI with or without IABP)
	(NCT04763200)
	2. REVIVED-BCIS2 (PCI + OMT vs PCI) (NCT01920048)
4. PCI in HFpEF	RCT or OS
5. Physiology-guided PCI in chronic HFrEF and HFpEF	RCT
6. Viability-guided PCI with cMR in chronic HFrEF	RCT
7. Complete vs incomplete PCI revascularization in HFrEF	RCT or OS
8. Prognosis of nonobstructive CAD in chronic HFrEF and HFpEF	Prospective multicenter OS
9. Mechanism of benefit of PCI in acute and chronic HFrEF	RCT or OS
	1. PROTECT IV (pVAD + HR-PCI vs. SOC PCI with or without IABP)
	(NCT04763200)
	2. STEMI-DTU (pVAD + PCI vs. SOC PCI)
	(NCT03947619)
10. Impact of frailty on acute and chronic PCI outcomes in HFrEF + HFpEF	RCT
	Prospective multicenter OS

cMR, cardiac magnetic resonance imaging; CS, cardiogenic shock; DanGer Shock, Danish-German Cardiogenic Shock; ECLS-SHOCK, extracorporeal life support in cardiogenic shock; HFpEF, heart failure with preserved ejection fraction; HFrEF, heart failure with reduced ejection fraction; HR-PCI, high-risk percutaneous coronary intervention; PCI, percutaneous coronary intervention; pVAD, percutaneous ventricular assist device; RCT, randomized controlled trial; OS, observational study; SOC, standard of care; STEMI-DTU, ST-Elevation Myocardial Infarction Door-to-Unload Trial.

Understanding the precise mechanisms through which PCI might benefit patients with HF is critical to both patient selection and PCI strategy. There is a paucity of data elucidating the potential mechanisms of benefit of PCI and its effect on myocardial recovery in patients with HF since these patients have been under-represented in clinical trials.^5^ In the Initial Invasive or Conservative Strategy for Stable Coronary Disease (ISCHEMIA) trial, patients with LVEF <35% or New York Association III or IV HF were excluded.^5^ A propensity matched analysis from the Duke Databank for Cardiovascular Disease of 901 patients with stable CAD and LVEF ≤35% undergoing PCI or medical therapy demonstrated no difference in the rate of all-cause mortality or cardiovascular hospitalization between therapies (hazard ratio: 1.18; 95% confidence interval: 0.96-1.44). Recent observational studies suggest that not only is PCI feasible in select patients with multivessel CAD and HF, but it may also be associated with reverse left ventricle (LV) remodeling and improved LVEF when performed with microaxial flow-mediated LV support and more complete revascularization.^11^ Whether PCI impacts the risk of sudden cardiac death and/or need for implantable cardioverter defibrillators in this patient population remains unknown.^12^ The Impella-Supported PCI in High-Risk Patients with Complex Coronary Artery Disease and Reduced Left Ventrticular Function (PROTECT IV, NCT04763200) should provide further insight into whether LV-assisted PCI improves outcomes, LV systolic function, and quality of life in high-risk patients with impaired LV systolic function and complex CAD.

Optimal patient selection and revascularization strategy for PCI in this context are particularly germane for patients with HF who are frail and older than 65 years, a rapidly growing segment of the population. Nearly 45% of patients with HF have frailty, and many are older adults.^13^ While a number of tools have been developed to risk stratify older frail patients with cardiovascular disease, many were intended for use in the outpatient setting and not specifically for PCI.^13^ Given the inextricable links between age, frailty, cardiovascular disease risk, and outcomes, the multidisciplinary heart team considering PCI in the older adult HF cohort needs to be armed with more effective risk stratification tools and a better understanding of the role of non-PCI therapies (physical, pharmacologic, nutritional, cognitive, and psychosocial).^13^

Ahmad et al. provide a contemporary review that calls attention to the multiple vagaries that exist within an important clinical problem that continues to vex practitioners. The most informative clinical trials of PCI for HF in the future will need to evaluate hard clinical outcomes (death, MI, cardiovascular and cerebrovascular events, and recurrent ischemia), identify mechanisms of benefit (ie, impact on ischemia, viability, and arrhythmia), and provide long-term follow-up of symptoms, quality of life, and HF hospitalizations. Given the complexities and risk/benefit trade-offs associated with PCI in this setting, patient shared decision-making must assume a central role and proceduralists should strive for “interventional virtuosity” by achieving complete revascularization, routinely using intravascular imaging/physiology, and taking specific measures to avoid bleeding, vascular complications, and acute kidney injury. Until further clinical trials guide us out of this “fog of therapeutic uncertainty,” clinicians must rely on available data as summarized by Ahmad et al. and our collective clinical experience to best inform therapeutic decisions.

## Declaration of competing interest

Dr Tehrani has received research grants from Boston Scientific and Inari Medical. He also serves a consultant to Boston Scientific, and he is on the advisory board for Abbott. Dr Damluji receives research funding from the Pepper Scholars Program of the Johns Hopkins University Claude D. Pepper Older Americans Independence Center (OAIC) funded by the National Institute on Aging P30-AG021334. Dr Batchelor serves as a consultant for Boston Scientific, Abbott, Medtronic, and V-Wave and receives research grant support from Abbott and Boston Scientific.

## Funding

This research did not receive any specific grant from funding agencies in the public, commercial, or not-for-profit sectors.
